# From bench to bedside: the promise and roadblocks of extracellular vesicle therapeutics

**DOI:** 10.7150/thno.131621

**Published:** 2026-02-26

**Authors:** Jing Zhang, Yuanwei Pan, Peng She, Lang Rao

**Affiliations:** 1 College of Food Science and Biology, Hebei University of Science and Technology, Shijiazhuang 050000, China.; 2 Institute of Chemical Biology, Shenzhen Bay Laboratory, Shenzhen 518132, China.; 3 Department of Orthopedics, The Seventh Affiliated Hospital of Sun Yat-Sen University, Shenzhen 518000, China.

**Keywords:** extracellular vesicles (EVs), clinical translation, single EV analysis, analytical techniques, machine learning

## Abstract

Extracellular vesicles (EVs) have transformed the landscape of precision therapeutics, owing to their intrinsic biocompatibility, capacity to transport biomolecules, and ability to cross biological barriers. Benefiting from these advantages, several EV-based therapeutic applications are currently under active development. However, most products remain at the preclinical stage due to significant translational challenges. In this review, we systematically outline these obstacles and highlight opportunities that can accelerate the clinical translation of EVs. We first identify core issues impeding clinical progress, such as difficulties in isolation, storage stability, precise characterization, biosafety evaluation, and quality control. After that, we examine emerging technologies designed to overcome these bottlenecks, including advanced bioreactors for large-scale production, microfluidic systems for high-purity separation, single-vesicle analysis technologies to address heterogeneity, and machine learning approaches that enhance standardization and support clinical decision-making. Finally, we discuss future directions focused on standardizing EV production, improving safety assessment, and ensuring regulatory compliance. By addressing current challenges and identifying promising pathways, this review aims to facilitate the translation of EV-based therapies from bench to bedside.

## 1. Introduction

Extracellular vesicles (EVs) are a class of biological nanoparticles secreted by cells and possessing a lipid bilayer membrane 1, 2. They can transport a variety of bioactive molecules, such as proteins, nucleic acids, lipids, and metabolites, between cells, tissues, organs, and even entire organisms, which plays critical roles in intercellular communication 2-4. These vesicles are categorized based on their biogenesis. Microvesicles arise from direct outward budding of the plasma membrane. Exosomes, however, are generated through endosomal trafficking: the plasma membrane invaginates to form an endosome, within which intraluminal vesicles assemble; these are then secreted when the endosome fuses with the plasma membrane 5, 6. Their sizes generally differ, with microvesicles measuring 30-150 nm and exosomes 100-1000 nm in diameter 5.

EVs exhibit pronounced heterogeneity not only in their cellular origin and biogenesis but also in size, shape, molecular cargo, and biological function 7, 8. This diversity stems from the wide range of parental cells, such as immune cells, neurons, stem cells, and tumor cells, and the distinct regulatory pathways governing EV formation and secretion, including post-translational modifications such as UBL3-mediated protein sorting to small EVs (sEVs), which significantly contributes to EV cargo heterogeneity 6, 9. Critically, the molecular makeup of EVs, including tetraspanins, integrins, growth factors, nucleic acids, and lipids, closely mirrors the physiological or pathological state of their source cells, rendering them valuable reservoirs of disease-specific biomarkers 10. However, this same heterogeneity presents significant challenges for the isolation, characterization, and standardization of EV-based analyses. Despite these technical hurdles, EVs possess inherent advantages as natural delivery platforms. Their biological origin enables efficient cargo transport, innate or modifiable targeting capabilities, and the ability to cross biological barriers such as the blood-brain barrier (BBB), often with fewer off-target effects than synthetic nanoparticles 11, 12. EVs also generally exhibit low immunogenicity and cytotoxicity, overcoming major limitations associated with conventional nanocarriers 13. Certain EV subtypes demonstrate natural tissue tropism, allowing them to home to specific cell types or sites of inflammation 14-16. The clinical potential of EV-based systems is exemplified by the licensed meningococcal outer membrane vesicle vaccine Bexsero®, which provides a benchmark for the translational development of EV therapeutics 17.

Due to these unique natural properties, EVs have become a hot topic in the development of therapeutic strategies and nanocarriers 18-26, particularly those derived from human mesenchymal stem cells (MSCs) 25, 27-31, adipose tissue 32, 33, or bone marrow 34, which have attracted great research interest and has been evaluated in both preclinical and clinical studies for diverse disease models. For instance, MSC-derived EVs (MSC-EVs) and some other EVs have been used as a novel therapy for the treatment of COVID-19 35-38. EV intervention can significantly reduce liver inflammation and fibrosis in diabetic animals, as well as markers of cerebrovascular-related damage, and improve multiple neurological function indicators such as cognition and movement 39-42. EVs can also can carry chemotherapy drugs or nucleic acid drugs, and by leveraging their inherent targeting properties to achieve precise therapy 43, 44. Given these advantages of EVs, some clinical applications of EV-based therapeutics are being actively developed (**Table 1**). However, most such products remain at preclinical stages, and there are many hurdles in clinical translation, such as poorly understood physiological roles, potential toxicity, and manufacturing challenges including scale-up, storage, and transportation.

In this review, we provide an overview of challenges and opportunities in the clinical translation of EVs. Firstly, we systematically outline the major challenges hindering the clinical translation of EVs, including difficulties in isolation, storage, characterization, biosafety assessment, and quality control. We then present emerging technologies designed to overcome these limitations, such as advanced bioreactors for scalable production, microfluidic systems for high-purity isolation, single-vesicle analysis for resolving heterogeneity, machine learning in standardization, and quality control for clinical decision support. These innovations collectively enhance the efficiency, functionality, and scalability of EV-based therapeutics, facilitating their transition toward clinical application. Finally, we discuss future directions and provide perspectives on the development of standardized, clinically compliant EV production and analytic platforms. We hope this review offers valuable insights for researchers aiming to advance EV technologies from bench to bedside.

## 2. Challenges in Clinical Translation

**Figure 1:**
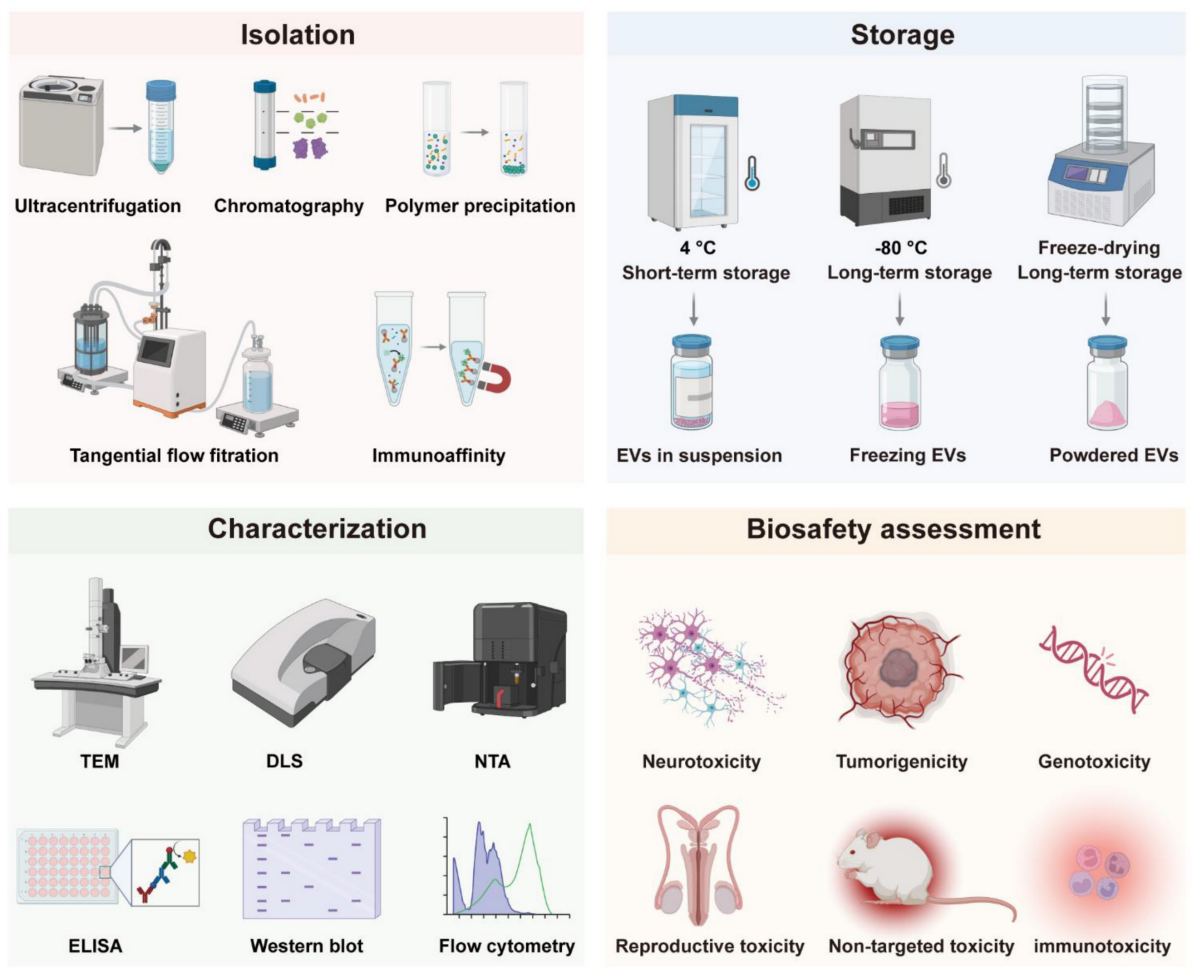
** Examples of traditional EV isolation, storage and characterization methods, and biosafety assessment indicators of EVs, illustrating the conventional workflow in extracellular vesicle research, which encompasses widely used isolation techniques, storage strategies, characterization approaches, as well as key biosafety evaluation endpoints essential for preclinical and therapeutic assessment**.

The clinical translation of EVs is confronted by several interconnected technical and regulatory hurdles that impede their transition from research tools to reliable therapeutics. Key challenges include the lack of scalable and standardized isolation techniques capable of ensuring high purity and yield, difficulties in maintaining structural and functional integrity during long-term storage, limitations in accurate characterization due to inherent heterogeneity, alongside complex biosafety profiling and stringent quality control requirements. Addressing these challenges is critical to achieving repeatability, safety, and compliance with clinical-grade production standards. In this section, we focus on these key strategies (**Figure 1**) and discuss their limitations in EV clinical translation (**Table 2**).

### 2.1 Isolation

Ultracentrifugation (UC) is the most widely reported isolation method. It efficiently recovers EVs from large volumes, such as cell culture supernatant, but co-isolates protein aggregates or heterogeneous vesicles, which compromises purity 45, 46. Furthermore, higher centrifugal forces can induce EV aggregation, membrane damage, and biomolecular damage 47, 48. Combining UC with size exclusion chromatography (SEC) can separate vesicles by size while preserving their biological integrity, thereby increasing purity. However, SEC may dilute the sample, necessitating a subsequent concentration step 49-51. Tangential flow filtration (TFF) presents a scalable and gentler alternative to UC, minimizing membrane fouling through tangential fluid flow 52. Nonetheless, TFF employs membrane surface filtration. During this process, high concentrations of proteins, lipoproteins, or contaminants can readily form gelatinous layers or occlude membrane pores, substantially reducing filtration efficiency. Alternatively, polyethylene glycol (PEG)-induced precipitation efficiently processes large volumes but yields impure EVs due to the co-precipitation of non-EV proteins 53. While the combination of UC and SEC offers decent yields, its specificity is limited by a reliance on overlapping physical properties. Therefore, orthogonal methods targeting independent physicochemical traits may improve selectivity, such as surface charge and size. For instance, anion exchange chromatography (AEC) can be coupled with SEC to isolate EVs from charged contaminants, though AEC requires optimization to prevent column-related losses 54, 55. Finally, affinity methods remain largely limited to small-scale applications due to their specificity for particular biochemical interactions 56.

As such, EV isolation requires large-scale cell culture and multiple purification steps, which are laborious and time-consuming. Furthermore, EVs are highly heterogeneous due to different cell types, culture conditions, isolation techniques, and inherent diverse subpopulations, making it difficult to produce homogeneous EVs. Regardless of the isolation method, the delivered EV population is always heterogeneous, invariably including some impurities, such as microvesicles, exosomes, and apoptotic bodies. These contaminants can affect the determination of EV dosing, as the current standard is to determine the dosage based on the number of EVs. Achieving high EV purity presents a pivotal challenge for clinical translation, with multistep isolation methods improving purity but often reducing yield. Most importantly, a universally accepted standard for EVs preparation is lacking, which seriously limits the high-throughput and large-scale production of EVs.

### 2.2 Storage

The longterm storage of EVs without loss of function is crucial for clinical transformation, as freshly isolated vesicles are often not readily available for immediate use. Studies have shown that EVs can be stored at 4 °C and -20 °C for brief periods, while long-term storage necessitates -80 °C conditions 57. Currently, the traditional method of preserving EVs is storage at -80 °C after the suspension of the EVs in saline or PBS buffer. Nevertheless, even at -80 °C, repeated freeze-thaw cycles and prolonged storage can also compromise the stability of EVs, leading to structural damage (EV rupture, fusion and aggregation) and content leakage, ultimately resulting in reduced therapeutic efficacy 58. Therefore, there is an urgent need for a more stable and effective preservation method.

Lyophilization, a well-established technique for stabilizing biological and pharmaceutical materials through controlled water removal, may be a promising method for the preservation of EVs 59. It allows for room-temperature storage of EV products and maintains functional biological activities of EVs, which can greatly improve the accessibility of EV therapy. However, EVs may have experienced freezing and dehydration stresses during freeze-drying, which can affect the efficacy of EVs 60. The addition of protectant containing sucrose and trehalose can effectively prevent EV aggregation, increase colloidal stability, and minimize sample loss 61-63. Moreover, in clinical applications, some practical problems need to be considered and solved. For example, the packaging of lyophilized EVs must be tailored to the administration route and transport conditions, including pressure humidity and temperature, while addressing barriers to therapeutic accessibility such as limited access to sterile water 60. As a relatively emerging field of study, there is limited existing data about freeze-drying of EVs, and additional studies are required to expand our understanding.

### 2.3 Characterization

Comprehensive characterization is essential for advancing EV research, which requires the integration of multiple analytical approaches to evaluate their physical and biochemical properties. Morphological and biophysical examination often involves high-resolution imaging techniques such as electron microscopy and atomic force microscopy (AFM) 64. These methods allow detailed visualization of EV size, distribution, and ultrastructure, though they often require complex sample preparation that risks introducing artifacts. For quantitative assessment of EVs in suspension, commonly used techniques include dynamic light scattering (DLS), nanoparticle tracking analysis (NTA), and tunable resistive pulse sensing (TRPS), which provide insights into hydrodynamic size, concentration, and surface charge 65-67. Nonetheless, each method has specific constraints: DLS demonstrates sensitivity toward larger particles within polydisperse samples, NTA necessitates high particle concentrations and careful optimization, and TRPS is vulnerable to pore blockage and signal interference. Biochemical characterization focuses on the molecular composition of EVs, including proteins, RNAs, and lipids. Assays for total protein or RNA content generally employ colorimetric, fluorometric, or electrophoretic methods; however, the accuracy of these measurements can be compromised by co-isolated contaminants. For the detection of specific proteins, techniques such as Western blotting, ELISA, and flow cytometry (FCM) are extensively utilized 68. Western blotting provides high specificity but is low in throughput and requires substantial sample amounts. ELISA offers greater throughput yet is prone to matrix effects in biologically complex samples. Although conventional FCM can detect protein markers on EVs, it still faces challenges in detecting sEVs (<200 nm). Importantly, the effectiveness of these conventional bulk analytical methods is fundamentally limited by the inherent heterogeneity of EV populations, which masks subpopulation-specific information and averages out critical functional variations.

EV heterogeneity refers to the substantial variability in physical and chemical properties among individual vesicles 69. This variability is broadly categorized into two main types: inter-subpopulation heterogeneity, describing differences between EVs from distinct sources, and intra-subpopulation heterogeneity, referring to variations among EVs from the same source.^7^ This inherent diversity underpins the wide functional versatility of EVs, influencing critical attributes such as cellular uptake, tissue targeting, and immunomodulatory potential 70, 71. However, it simultaneously presents a major translational challenge, hindering reproducible characterization, standardization, and clinical application. On a physical level, heterogeneity is evident in a broad size distribution (approximately 30 to over 150 nm), which dictates cargo capacity, and in a range of densities, although the functional implications of density variation are not fully understood 72-74. At the molecular level, heterogeneity manifests in variable expression of key surface proteins, including tetraspanins like CD9, CD63, and CD81 that govern cellular interactions, and in highly diverse luminal cargo comprising proteins, nucleic acids, and metabolites 75. A critical knowledge gap remains in precisely mapping how specific surface markers correlate with internal payloads at the single-vesicle level. This lack of understanding obscures clear structure-function relationships and significantly complicates the rational design of EV-based diagnostics and therapeutics.

### 2.4 Biosafety Assessment

The general principles for safety evaluations of EV drugs are based on the technical guidelines for the Research and Evaluation of Cell and Gene Therapy Products and comply with Good Laboratory Practice (GLP) guidelines. Studies conducted under non-GLP conditions should be evaluated for reliability and impact on overall safety assessment 76. Both general toxicity tests and personalized safety evaluations must be established based on EV sources. Key safety concerns include neurotoxicity, tumorigenicity, genotoxicity, reproductive toxicity, immunotoxicity, and immunogenicity 77. EVs can cross the blood-brain barrier, exhibiting neuroprotective or neuroinflammatory effects depending on their origin, but long-term neurological impacts require further study 78, 79. While EVs are non-living and theoretically non-tumorigenic, their bioactive components such as noncoding RNAs necessitate tumorigenicity assessments, particularly for long-term or repeated dosing 80. Genotoxicity risk is low since EVs do not integrate into the host genome, but engineered EVs with novel components require genetic toxicity testing 81. Reproductive toxicity studies suggest EVs may be protective such as MSC-EVs in ovarian failure models, though long-term effects remain unclear. Immunomodulation is a key therapeutic function of EVs, but immunogenicity depends on source and surface modifications, requiring careful evaluation of antigenicity, especially for immune cell-derived or engineered EVs. Comprehensive toxicokinetic analysis is currently limited by technological constraints, necessitating risk-based approaches with plasma concentration data from repeat-dose studies. Overall, EVs particularly stem cell-derived demonstrate low safety risks, but long-term, cross-species studies are needed to confirm safety across therapeutic applications. Future research should focus on toxicity mechanisms and long-term monitoring to ensure clinical safety. For EV drugs classified as gene therapies, additional reference should be made to gene therapy product guidelines 82, 83.

### 2.5 Quality Control and Regulatory Landscape

Quality control is a critical component of EV drug development and evaluation, encompassing the entire manufacturing process under cGMP principles to ensure high-quality production by trained personnel in controlled facilities with detailed documentation 76, 84. Although EV drug development may reference guidelines for cell and gene therapy products, the distinct physicochemical properties and biological activities of EVs compared to parental cells necessitate unique manufacturing processes, requiring a comprehensive quality control system covering production materials, process parameters, process controls, release testing, stability studies, and process validation 85. Rigorous supplier qualification is essential for production materials such as raw materials, excipients, consumables, and packaging materials, with particular attention to viral and immunogenicity risks associated with animal-derived components, while engineered EVs additionally require strict quality control of genetically modified materials like plasmids and viruses 53, 86. Quality standards must be established through risk assessment, covering characteristics, purity, safety, and efficacy while being dynamically optimized throughout development, with stability studies following cell therapy product requirements to verify stability under various storage and transportation conditions 87. Process validation requires demonstrating reproducibility across three consecutive production batches, and since EVs cannot undergo terminal sterilization, microbiological safety controls must be strengthened throughout production 76. Ultimately, EV drugs require an end-to-end quality control strategy integrated across the entire R&D lifecycle to ensure safe and effective clinical application through standardized manufacturing and rigorous quality oversight.

However, the clinical advancement of EV-based therapeutics is encountering a complex and evolving regulatory landscape, which presents several pivotal challenges to their standardized development and approval. A primary issue lies in the complex and ambiguous classification pathways. EV-based drugs possess a dual identity, functioning both as intrinsic therapeutic entities and as engineered delivery vehicles. Regulatory agencies such as the United States Food and Drug Administration (U.S. FDA) and European Medicines Agency (EMA) generally classify natural therapeutic EVs as biological products, whereas EVs serving as carriers for nucleic acids or other active molecules may be categorized as gene therapy products or advanced therapy medicinal products, depending on the nature of the cargo 88, 89. This classification ambiguity complicates development pathways and regulatory expectations. Furthermore, there is a notable absence of EV-specific technical guidelines from major global health authorities. While existing frameworks for cell and gene therapies offer some reference, they fail to address EV-unique parameters such as vesicle heterogeneity, membrane-based structure, and complex cargo profiles 90. This guideline gap extends to critical areas including quality control, nonclinical safety assessment, and potency testing, where standardized methods for evaluating purity, impurity profiles, biological activity, and pharmacokinetics of EVs are still underdeveloped 91. Consequently, the field urgently requires the establishment of tailored regulatory standards and robust analytical tools to ensure the safety, efficacy, and consistency of EV-based therapeutics as they advance through clinical development.

## 3. Opportunities from Advanced Technology

**Figure 2:**
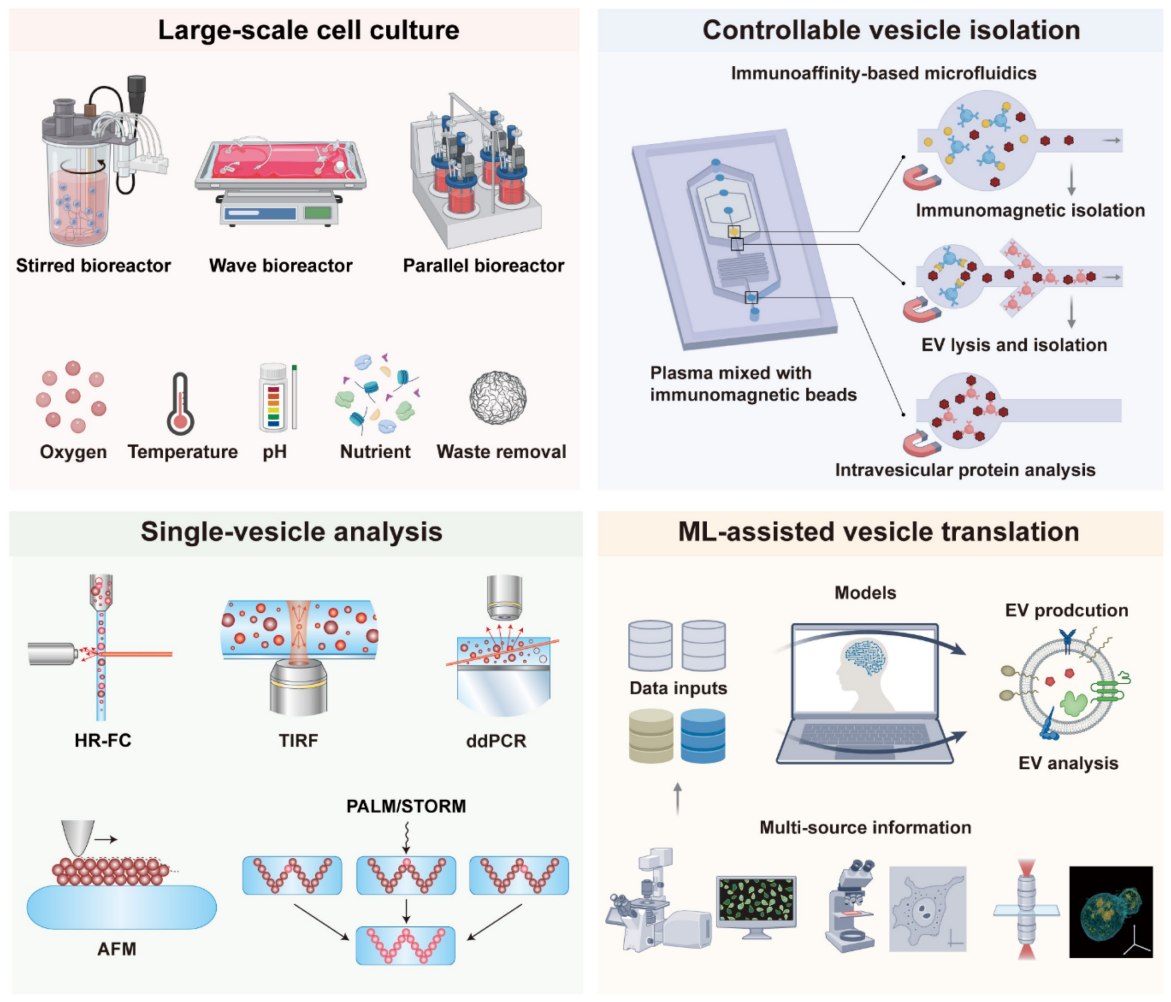
** Representative advanced techniques to promote clinical translation of EVs, including bioreactors for large-scale cell production, microfluidic chips for EV controllable isolation, single-vesicle analysis techniques to overcome heterogeneity, and machine learning to assist clinical translation**.

As mentioned previously, traditional production and analysis methods of EVs still have many problems, which pose a significant challenge to the clinical translation of EVs. To address these challenges, numerous advanced technologies have been developed, including bioreactors for large-scale cell production, microfluidic chips for EV isolation, single-vesicle analysis techniques to overcome heterogeneity, and machine learning to facilitate clinical translation (**Figure 2**). These advanced techniques significantly improve the production efficiency, purity, and scalability of EVs, making them more suitable for clinical applications. This section provides an overview of recent advances in EV clinical applications with these advanced technologies.

### 3.1 Bioreactor Technology

Bioreactors provide a key solution to the limitations of conventional EV production methods including two-dimensional adherent cultures static systems and small-scale shake flask cultivation 92-94. These traditional approaches are associated with low yield poor scalability substantial batch-to-batch variability and high costs. By enabling precise control over critical culture parameters such as temperature, pH, dissolved oxygen, nutrient supply and metabolic waste removal, bioreactors can facilitate high-density cell culture while maintaining a stable microenvironment 95. Such control facilitates the production of EVs with enhanced functions, including improved immune regulation, anti-inflammatory effects, tissue repair capabilities, or targeting specificity 96. This approach significantly enhances EV secretion efficiency overall yield and batch consistency establishing bioreactors as an essential technological platform for large-scale standardized and reproducible EV manufacturing. Such advancements are pivotal for accelerating the translation of EV research from benchside to clinical and industrial applications.

**Figure 3:**
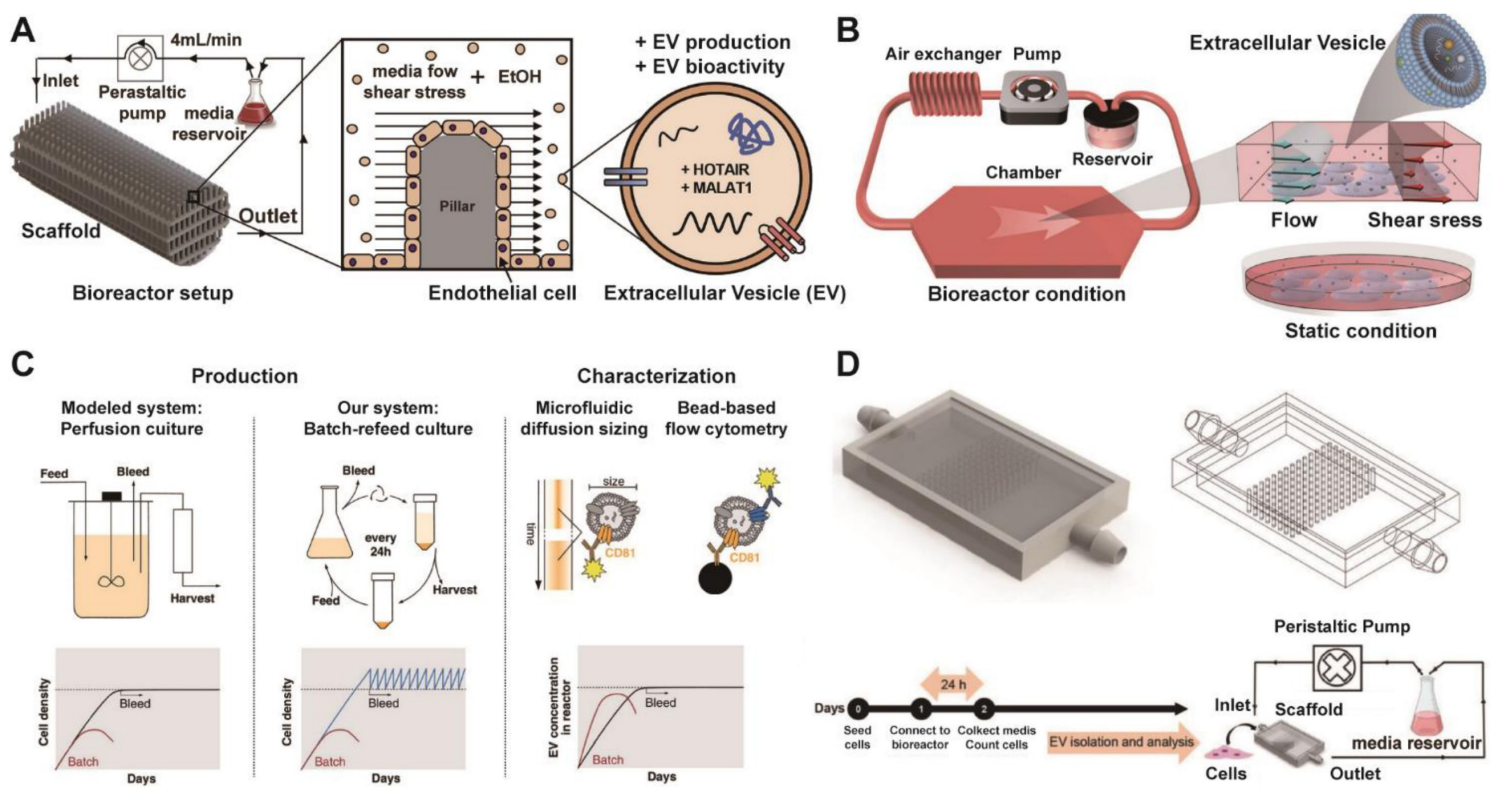
** Integrated platform of advanced bioreactor technologies for optimized EV production.** (A) Diagram of the 3D-printed scaffold perfusion bioreactor system for production of endothelial cell-derived EVs. Adapted with permission from 98, copyright 2019 Elsevier. (B) Diagram of the closed-loop perfusion bioreactor system applying fluid-derived shear stress to enhance EV production, versus a static control. Adapted with permission from 99, copyright 2022 Wiley. (C) Diagram illustrating the batch-refeed culture system used to mimic perfusion bioreactors for EV production, involving periodic cell resuspension in fresh medium to maintain high density, and subsequent EV quantification via CD81/CD63 bead-based flow cytometry. Adapted with permission from 100, copyright 2023 Wiley. (D) Schematic of the scaffold-based perfusion bioreactor system, showing the internal pillar design, experimental timeline, and the operational setup with media circulation via a peristaltic pump at controlled flow rates. Adapted with permission from 101, copyright 2023 Wiley.

Currently, bioreactors for EVs mainly include stirred bioreactors, which are suitable for suspending cells or enabling high-density culture of adherent cells on microcarriers and are easy to scale up; these systems serve as the mainstream choice for industrialization 97. Compared to conventional 2D culture, 3D-printed scaffold-perfusion bioreactor system can significantly enhance the production of EVs from human endothelial cells, achieving over a 100-fold increase in yield (**Figure 3A**) 98. Furthermore, the flow-induced intracellular calcium increase drives a sevenfold enhancement in MSC-EV production within a flat-plate perfusion bioreactor compared to static culture (**Figure 3B**) 99. Paganini et al. also demonstrated that perfusion-mimicking cultures address the critical challenges of EV manufacturing by enabling a substantial increase in yield while ensuring consistent production of specific EV subpopulations (**Figure 3C**) 100. Besides, the integration of 3D-printed scaffolds within a perfusion bioreactor is presented as an effective strategy to achieve high-yield production of potent MSC-EVs for therapeutic applications (**Figure 3D**) 101. In addition to, airlift bioreactors utilize gas circulation to achieve mixing and oxygen transfer, generate low shear force, and are ideal for cells sensitive to shear stress 102. Hollow fiber bioreactors provide an extremely large surface area for cell growth, support long-term high-density culture of adherent cells, and contribute to increased EV yields 103. Packed bed bioreactors enable cells to grow attached to a fixed carrier, making them suitable for high-density cultivation 104. These approaches represent a mainstream strategy for large-scale production. Shake flasks and rotating flasks are suitable for early process development and small-scale experiments, but they are not easily scalable.

When implementing bioreactors for scaled-up EV production, several practical considerations require attention. First, process parameters that are optimized at laboratory scale, such as shear stress, oxygen transfer and nutrient gradients, often do not translate linearly to industrial-scale reactors, necessitating careful re-optimization to maintain EV yield, quality, and consistency. Second, the capital and operational costs of GMP-compliant bioreactor systems are substantial and must be factored into process economics. Furthermore, the bioreactor configuration and culture parameters must be tailored to the specific producer cell type, demanding dedicated process development. Finally, attention must be paid to how the dynamic bioreactor environment itself might influence the resulting EV secretome profile. Therefore, successful translation relies on rigorous process optimization, monitoring using process analytical technology, and validation to ensure that the EVs produced at scale are both therapeutically consistent and economically viable.

### 3.2 Controllable Vesicle Isolation Technology

The clinical translation of EVs requires the development of controllable, reproducible, and scalable isolation technologies. Such development demands that these advanced platforms guarantee batch-to-batch consistency, functional integrity, and compliance with regulatory standards. The following sections highlight some advanced isolation technologies, including microfluidic systems, hydrogel-based matrices, and bio-orthogonal chemistry approaches, which opens new avenues for controllable EV separation.

#### 3.2.1 Microfluidics

Microfluidic-based EV isolation strategies mainly include hydrodynamic-based methods, size-based methods, immunoaffinity-based methods, and physical field-driven techniques 105-107. Compared with the traditional large-volume centrifugation or precipitation methods, these approaches have significant advantages such as low sample consumption, fast isolation speed, high integration level, ease of miniaturization and automation, which is an important frontier direction in the current field of EV research.

**Figure 4:**
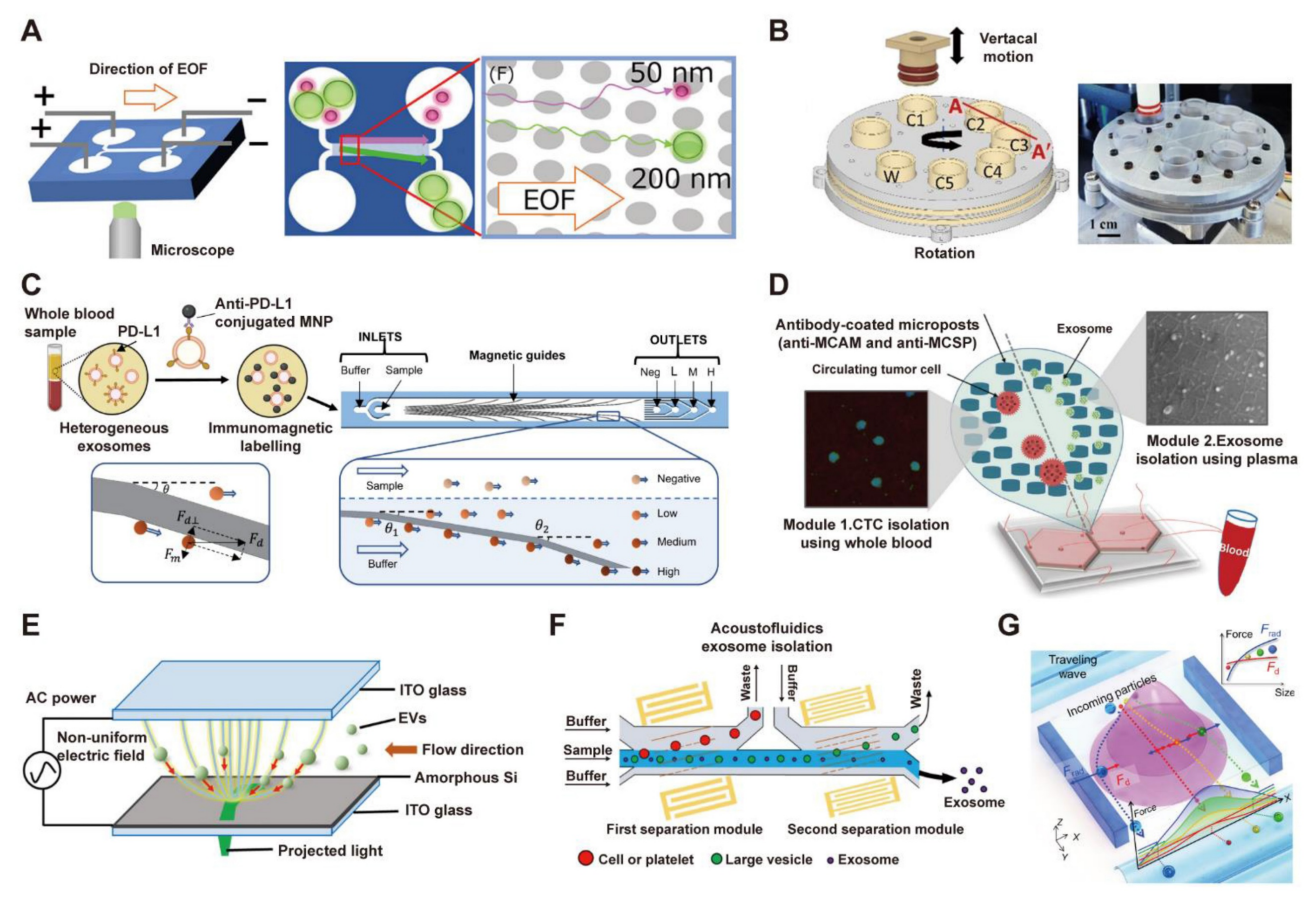
** Microfluidic technologies for EV isolation employing dielectrophoresis, acoustofluidics, immunoaffinity, and size-based filtration.** (A) EOF-derived nano-DLD chips feature a column array containing nanoscale gaps. Adapted with permission from 110, copyright 2019 American Chemical Society. (B) Schematic and photograph of an automated system for size-selective filtration of EVs, showcasing its configuration of filtration chambers (C1-C5: 200, 100, 80, 50, 30 nm pores) and a waste outlet (W). Adapted with permission from 111, copyright 2022 The Royal Society of Chemistry. (C) Immunomagnetic labeling of plasma sEVs with PD-L1-specific MNPs, followed by their sorting in a microfluidic chip via magnetic deflection, enables phenotypic profiling of heterogeneous exosomes. Adapted with permission from 116, copyright 2023 Springer Nature. (D) Schematic of the immunoaffinity-based microfluidic chip for concurrent isolation of circulating tumor cells (CTCs) and exosomes from blood samples using antibody-functionalized microposts. Adapted with permission from 117, copyright 2020 Wiley. (E) Optically induced dielectrophoresis (ODEP) was employed for size-based EV isolation. This technique exploited dynamic light patterns to generate an ODEP force that attracted EVs, with their size-based sorting achieved by optimizing the light intensity and scanning velocity. Adapted with permission from 120, copyright 2024 The Royal Society of Chemistry. (F) An acoustofluidic chip with two separation modules for the size-based isolation of exosomes from plasma, removing cells/platelets in the first module and larger vesicles in the second, resulting in a purified exosome sample. Adapted with permission from 121, copyright 2024 Springer Nature. (G) Schematic depicting the dynamics of nanoparticle separation in an acoustic virtual wave-pillar field. Frad, acoustic radiation force; Fd, drag force. Adapted with permission from 122, copyright 2022 American Association for the Advancement of Science, AAAS.

The hydrodynamic conditions within microfluidic channels can be precisely engineered to exploit specific fluid dynamics, allowing for the size- and density-based separation of particles such as EVs in a label-free manner. Utilizing the deterministic lateral displacement (DLD) principle, EVs and other particulates follow distinct migration trajectories depending on their dimensions, enabling efficient fractionation without the need for labels 108, 109. In a recent advance, Hattori et al. described a DLD-based microfluidic chip for EV isolation that employs electroosmotic flow (EOF) rather than high-pressure pumping, which can reach over 200 kPa in nano-DLD configurations, to enable gentle handling and effective separation of EVs within nanofluidic environments (**Figure 4A**) 110.

Size-based isolation methods exploit the size differences between EVs and other components to achieve physical isolation 105. Common strategies include three key approaches: using nanochannel or microsieve filtration to retain larger impurities while allowing EVs to pass through; employing deterministic lateral displacement to separate particles by their regular deflection within micropillar arrays; and utilizing microfluidic size exclusion technology to enrich EVs through size-ordered particle elution. For example, Seder et al. introduced an automated system for size-based fractionation of EVs, as illustrated in **Figure 4B** 111. The device used a vertical plunger and a multi-chamber chip with nanoporous filters to size-selectively isolate EVs (30-200 nm) through sequential filtration. This approach achieved an 89% EV recovery rate and a tenfold improvement in purity over ultracentrifugation.

Immunoaffinity capture methods utilize the specific binding of EV surface proteins to antibodies or aptamers immobilized on the chip surface to achieve highly selective capture and isolation of target EVs 105, 112-115. Chen et al. reported a magneto-activated nanoscale cytometry platform (NanoEPIC) for the high-throughput phenotypic sorting of sEVs. This platform leverages immunomagnetic labeling in conjunction with a microfluidic device containing embedded ferromagnetic guides to deflect sEVs based on their surface marker expression levels (**Figure 4C**) 116. Additionally, Kang et al. introduced a dual-isolation microfluidic device (DUO) functionalized with melanoma-specific antibodies (MCAM/MCSP) to simultaneously capture circulating tumor cells (CTCs) and tumor-derived exosomes from a single blood sample of melanoma patients (**Figure 4D**) 117. These method offers strong specificity and high enrichment efficiency, but it also presents challenges such as high antibody cost, interference from non-specific adsorption, and chip stability issues caused by biocontamination.

Physical field-driven isolation methods exploit the responsiveness of EVs to external physical fields to achieve efficient, gentle isolation while maintaining activity in a contactless, label-free manner 48, 118, 119. For example, Soong et al. leveraged the highly flexible and precise electric field control of optically induced dielectrophoresis (ODEP) for efficient, size-based isolation of EVs (**Figure 4E**) 120. In the work of Wang et al., two surface acoustic wave (SAW) modules were integrated into a microfluidic chip to isolate exosomes from plasma with high purity. The device uses a 20 MHz wave to remove cells and platelets, followed by a 40 MHz wave to eliminate larger EVs, thereby enriching intact exosomes (30-150 nm) and showcasing the tunable resolution of SAWs for size-based separation (**Figure 4F**) 121. Another example is the ANSWER platform, which employs arrays of virtual acoustic wave pillars to achieve high-resolution fractionation of sEV subpopulations. This method has been demonstrated to enable single-step, high-purity isolation of exomeres and exosomes directly from plasma within minutes, presenting a powerful tool for sEV-based liquid biopsies (**Figure 4G**) 122.

Microfluidic technologies provide notable advantages in purity and analytical resolution, several practical constraints hinder their direct translation to clinical-scale EV manufacturing. The limited throughput of most microfluidic devices, which operate at microliter scales suitable for diagnostics, is inadequate for the liter-scale volumes required in therapeutic manufacturing. Fabrication costs and technical complexity are also substantial, especially for chips with integrated sensors. Additionally, operational issues like channel fouling and long-term stability must be addressed for reliable use in controlled manufacturing. Thus, while microfluidics holds clear value for diagnostics and process analytics, its integration into end-to-end, large-scale EV manufacturing is currently limited by bottlenecks in throughput, scalability, and cost. Overcoming these hurdles requires coordinated innovations in chip design, system integration, and process economics.

#### 3.2.2 Hydrogels

**Figure 5:**
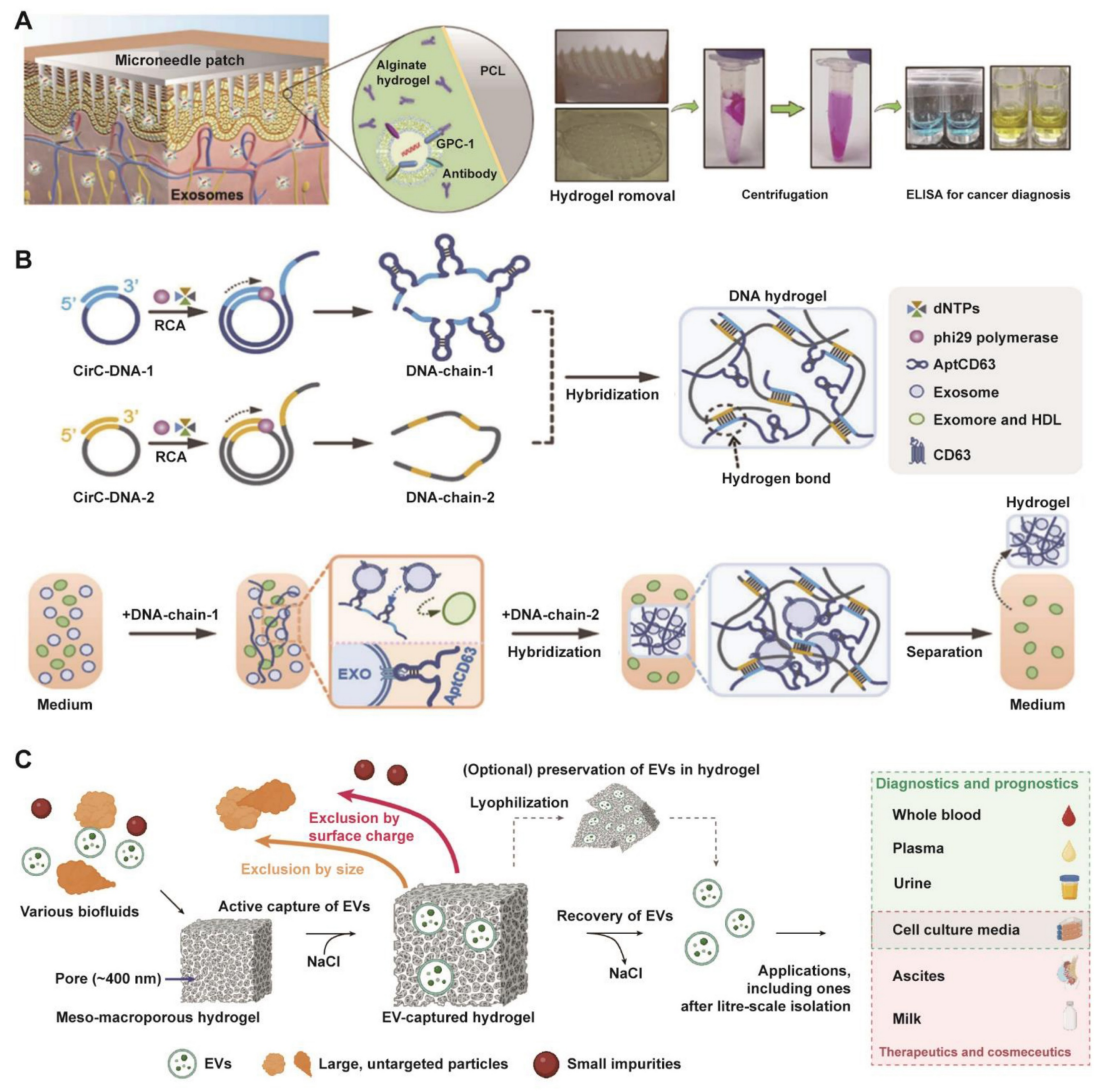
** Multifunctional hydrogel systems enabling advanced EV isolation.** (A) The volume-swellable hydrogel-coated microneedles enable rapid extraction of EVs from skin interstitial fluid for early cancer diagnosis. Adapted with permission from 126, copyright 2023 American Chemical Society. (B) A viscoelasticity-tunable DNA hydrogel for the specific capture and isolation of exosomes, leveraging a CD63 aptamer-functionalized rolling circle amplification (RCA) framework. Adapted with permission from 127, copyright 2023 National Academy of Sciences. (C) Schematic of a meso-macroporous hydrogel for the direct isolation of EVs from various biofluids and its subsequent versatile applications. Adapted with permission from 128, copyright 2025 Springer Nature.

A variety of new materials have demonstrated unique advantages in the field of EV research. Among these materials, hydrogels 123-125, with their tunable three-dimensional porous networks and dynamic swelling and contraction in response to stimuli, present a versatile platform for EV isolation. Based on these properties, Park et al. reported the utilization of a swellable hydrogel to extract EVs from interstitial fluid (**Figure 5A**) 126. Building on this concept, they developed a microneedle patch functionalized with GPC-1 antibody, which enabled the specific isolation and subsequent ELISA quantification of tumor-derived EVs for the non-invasive diagnosis of colorectal cancer. Additionally, Tang et al. designed a bifunctional DNA hydrogel where one DNA chain formed a porous network for EV encapsulation while the other provided specific targeting (**Figure 5B**) 127. This system leveraged the hydrogel with optimal viscoelasticity for rapid, equipment-free isolation and a strand-displacement mechanism for the intact release of EVs, offering a versatile platform for gentle biomarker purification. In the latest study, researchers also developed a meso-macroporous hydrogel-based platform for direct isolation of exosomes without pretreatment and were able to scale up the amount of material required to several liters (**Figure 5C**) 128. It achieved unique pore characteristic through cryo-photocrosslinked PEG700DA matrices and exploited salt-modulated EV aggregation for charge-selective capture and efficient recovery. This technology achieves 1,539× higher yield than ultracentrifugation for milk EVs, while also serving as a solid-phase matrix for preserving EVs for on-demand downstream analyses.

#### 3.2.3 Click chemistry

**Figure 6:**
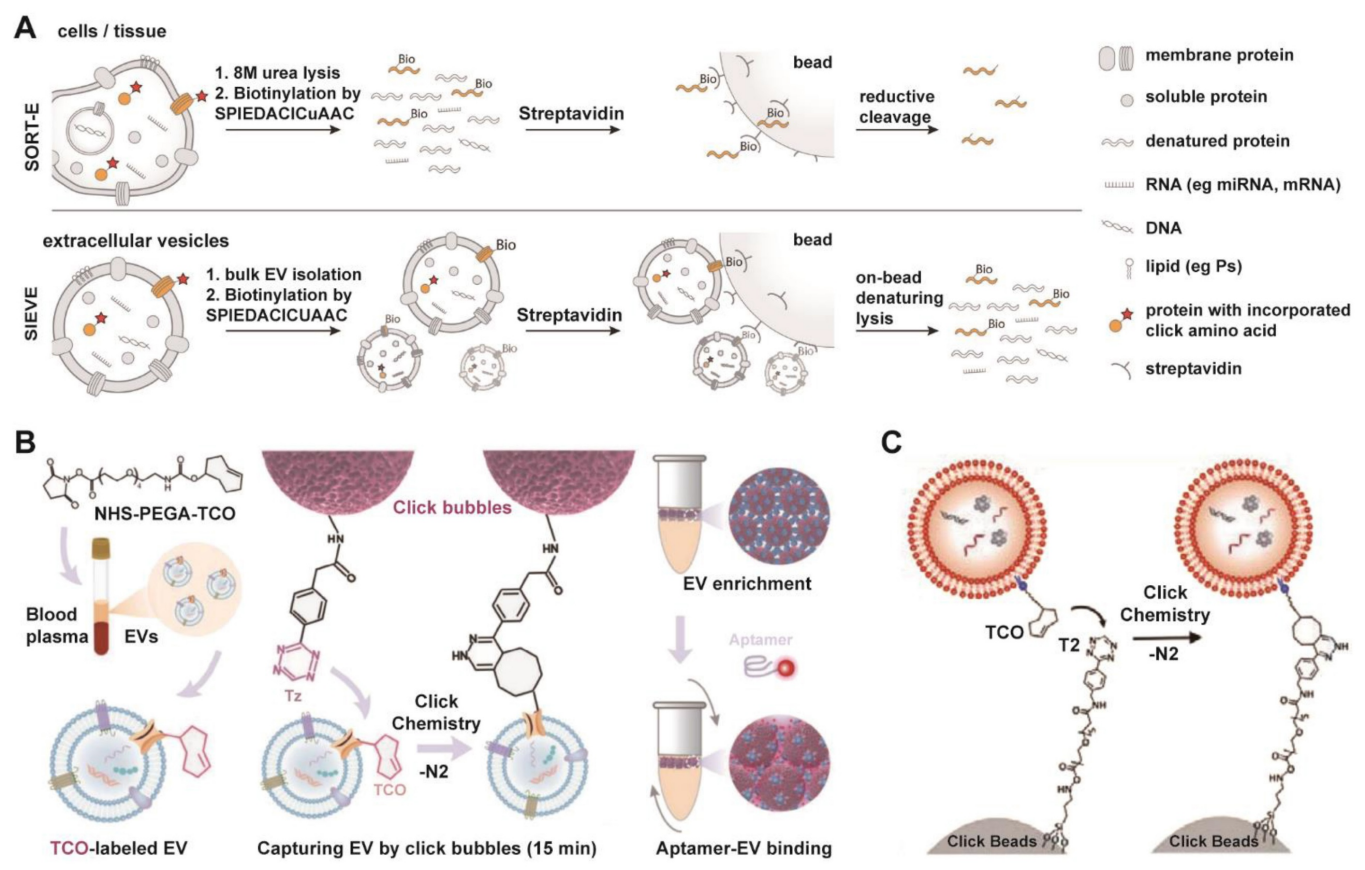
** Modular click chemistry strategies enabling rapid and efficient EV isolation, utilizing bioorthogonal conjugation for capture, enrichment, and downstream analysis.** (A) Schematic of the SIEVE technology for cell-of-origin selective EV enrichment, which utilizes bioorthogonal protein labeling via engineered tRNA synthetase/tRNA pairs to incorporate non-canonical amino acids, enabling specific capture of intact extracellular vesicles and co-purification of their associated cargo. Adapted with permission from 133, copyright 2023 National Academy of Sciences. (B) Schematic of EV enrichment and phenotyping using aptamer-functionalized click bubbles, demonstrating rapid capture via click chemistry and subsequent fluorescent aptamer binding. Adapted with permission from 134, copyright 2024 Wiley. (C) Efficient EV capture through a bioorthogonal click reaction between TCO-tagged EVs and tetrazine-functionalized Click Beads. Adapted with permission from 135, copyright 2022 Wiley.

EV engineering leverages chemical biology tools, such as bio-orthogonal click chemistry, a class of selective, rapid, and biocompatible reactions that proceed in living systems without interfering with native biological processes, to modify EV functions with superior precision and efficiency 129-132. This approach enables the targeted augmentation of inherent properties and the programmable grafting of exogenous capabilities. Furthermore, the application of bio-orthogonal click chemistry has emerged as a powerful and modular strategy for the rapid and efficient isolation of EVs, addressing key limitations of conventional methods. An example is the SIEVE platform (**Figure 6A**), which achieves cell-of-origin selective EV enrichment 133. This technology employs engineered tRNA synthetase/tRNA pairs to incorporate non-canonical amino acids into newly synthesized proteins of target cells, enabling subsequent bioorthogonal labeling and highly specific capture of intact EVs along with their associated molecular cargo 133. Beyond selective isolation, click chemistry also facilitates rapid EV capture from complex biofluids. As illustrated in **Figure 6B**, aptamer-functionalized click bubbles can efficiently capture TCO-labeled EVs within 15 min via a tetrazine-Trans-Cyclooctene (TCO) reaction, allowing for subsequent phenotyping through fluorescent aptamer binding 134. Furthermore, the strategy can be simplified for highly efficient bulk enrichment, where TCO-tagged EVs are directly captured by tetrazine-functionalized solid supports, termed “Click-Beads” (**Figure 6C**) 135.

While click chemistry offers remarkable specificity and efficiency, its clinical translation for EV isolation or engineering must carefully consider biocompatibility and immunogenicity. The introduction of non-natural chemical groups (e.g., azides, cyclooctynes) onto EV surfaces or the use of synthetic linker molecules could potentially alter the EV's native interaction with the immune system. Modified EVs might be recognized as foreign, triggering unintended immune responses or accelerating clearance *in vivo*, which could diminish therapeutic efficacy or cause adverse effects. Therefore, rigorous assessment of the immunogenic potential of click chemistry-modified EVs is essential in preclinical development. Future work should focus on optimizing reaction conditions to minimize surface alteration and developing novel bio-orthogonal pairs with even greater efficiency and biocompatibility for *in vivo* applications.

### 3.3 Single-vesicle Analytical Technology

In traditional research, we were often limited to conducting overall analyses of EV populations and had difficulty distinguishing the unique characteristics and behaviors of different subgroups of EVs. The inherent heterogeneity of EVs poses a significant challenge to their clinical application, as bulk analysis methods often obscure critical disease-specific signatures. To address this, a series of single-vesicle analysis platforms has emerged, enabling the multiplexed profiling of individual EVs at unprecedented resolution.

#### 3.3.1 Single-vesicle Analysis

Single vesicle analysis technology is a rapidly developing high-resolution research method that can reveal the physical properties, chemical composition and biological functions of individual vesicles at the single-vacuole level 136. These technologies are mainly based on different principles such as optics, electronics, and mechanics, each with its own unique features and strong complementarity.

**Figure 7:**
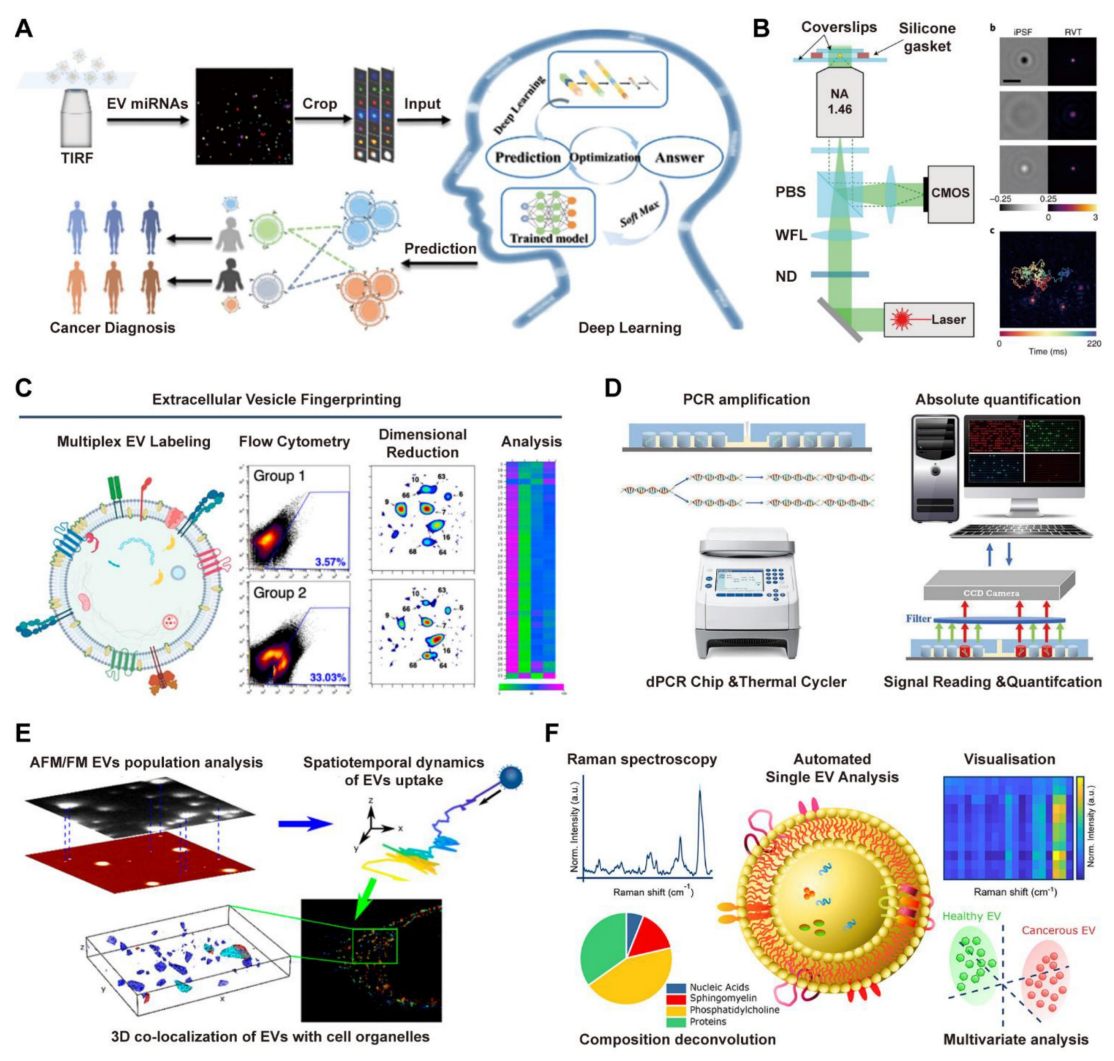
** Advanced single-vesicle profiling technologies for decoding EV heterogeneity.** (A) Principle of Multi-miRNA TIRF Imaging and Deep Learning from Automated Single-EV Analysis to Diagnostic Classification. Adapted with permission from 137, copyright 2024 American Chemical Society. (B) Configuration and tracking capability of the iSCAT microscopy system, featuring the optical path design, point spread function demonstration, and single-nanoparticle trajectory analysis. Adapted with permission from 138, copyright 2022 Springer Nature. (C) Principle of EV Fingerprinting for resolving heterogeneity, employing dimensional reduction of multiparametric single-EV flow cytometry data based on membrane order and size to discern distinct vesicle populations and cargo distribution. Adapted with permission from 142, copyright 2024 American Chemical Society. (D) Schematic illustrating the core workflow of the dPCR chip for absolute quantification of tumor-associated lncRNAs in salivary extracellular vesicles, encompassing PCR amplification in partitioned microchambers, thermal cycling, and subsequent fluorescence signal reading. Adapted with permission from 143, copyright 2023 Elsevier. (E) Single-molecule microscopy characterization of HEK293T-derived EVs, combining AFM, fluorescence imaging, and dSTORM to determine size distribution, uptake dynamics, and endosomal colocalization in HeLa cells. Adapted with permission from 144, copyright 2023 American Chemical Society. (F) Schematic of the SPARTA system for high-throughput, label-free analysis of EVs, utilizing Raman spectroscopy and dimensional reduction analysis (DRA) to achieve highly accurate classification of cancer EVs and breast cancer subtypes. Adapted with permission from 145, copyright 2021 American Chemical Society.

In optical imaging, super-resolution microscopy techniques overcome diffraction limits (~20-50 nm) via fluorescent molecule localization, key for nanoscale vesicle surface protein analysis and live-cell dynamics tracking. These methods have become indispensable tools for investigating nanoscale surface protein distributions on vesicles and tracking individual vesicle dynamics in living cells. For instance, Total Internal Reflection Fluorescence (TIRF) microscopy uses evanescent waves (100-200 nm surface excitation) to reduce background and enables multi-miRNA imaging on single EV surfaces (**Figure 7A**) 137, while interferometric Scattering (iSCAT) microscopy detects scattered light interference and tracks individual vesicle trajectories in real time with millisecond resolution (**Figure 7B**) 138.

Flow cytometry serves as a fundamental tool for single vesicle analysis. High resolution flow cytometers, especially nanoflow cytometry systems with detection limits reaching 70-100 nm, enable simultaneous assessment of vesicle size distribution and surface marker expression through multicolor fluorescent labeling 139-141. For instance, the EV Fingerprinting method applies dimensional reduction to flow cytometry data using parameters such as membrane order and size to resolve distinct vesicle subpopulations (**Figure 7C**) 142. In molecular analysis, digital droplet Polymerase Chain Reaction (ddPCR) platforms provide absolute quantification of vesicle derived RNA content. By isolating single EVs into discrete microchambers or droplets, these systems facilitate nucleic acid amplification and enable precise Poisson based counting of target RNA molecules, including tumor associated transcripts from salivary EVs (**Figure 7D**) 143.

Electrical and spectroscopic techniques provide critical Technologies for single-vesicle analysis, complementing optical and molecular approaches. Atomic force microscopy (AFM) offers detailed topographical imaging and quantifies mechanical properties such as elasticity and adhesion through force spectroscopy, providing direct insight into structure-function relationships of vesicles. As demonstrated in an integrated microscopy approach (**Figure 7E**), AFM combined with super-resolution techniques such as direct stochastic optical reconstruction microscopy (dSTORM) enables comprehensive characterization of EV morphology, cellular uptake, and intracellular localization 144. In parallel, Raman spectroscopy, particularly surface-enhanced Raman scattering (SERS), enables label-free detection of molecular vibration fingerprints to reveal intravesicular chemical composition. Leveraging this principle, the SPARTA system (**Figure 7F**) integrates Raman spectroscopy with dimensional reduction analysis for the accurate and label-free classification of EVs, proving highly valuable for identifying disease-specific vesicle signatures 145.

#### 3.3.2 High-throughput Platforms

**Figure 8:**
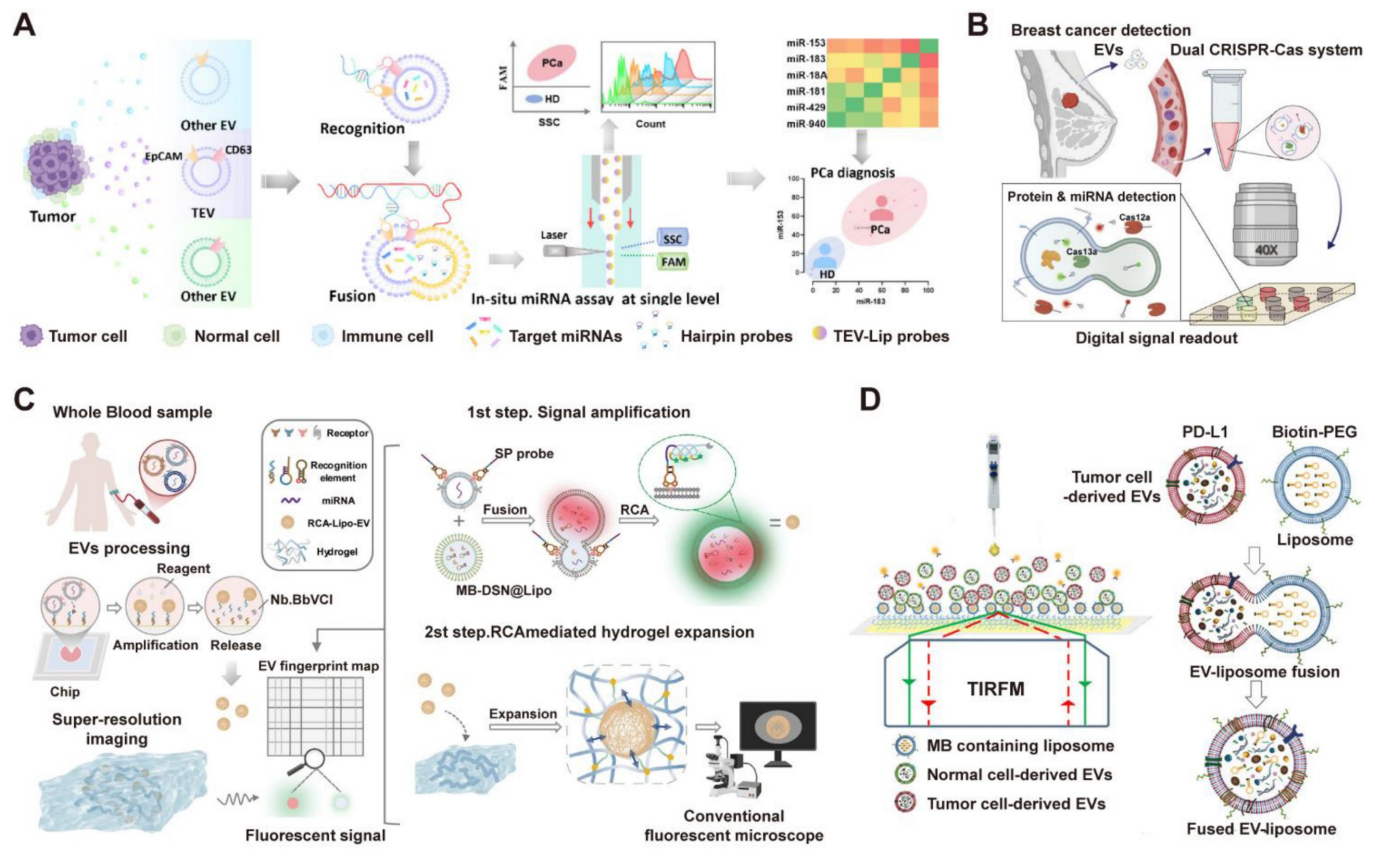
** High-throughput single-vesicle platforms resolve EV heterogeneity through multiplexed profiling at single-particle resolution.** (A) The high-throughput miR-nSTEV platform enables precise prostate cancer diagnosis by specifically capturing tumor extracellular vesicles (TEVs) and performing in-situ, single-vesicle miRNA profiling via nanoflow cytometry. Adapted with permission from 146, copyright 2025 American Chemical Society. (B) Through co-encapsulation in microwells, the ddSEE assay facilitates concurrent protein (Cas12a, red) and miRNA (Cas13a, green) detection in single EVs, triggered by liposome fusion and antibody-DNA activation, followed by digital fluorescence imaging. Adapted with permission from 147, copyright 2025 American Chemical Society. (C) Integrating in-situ protein and miRNA detection via rolling circle amplification (RCA) and liposome fusion, the RCA-ExM method enables high-content single-EV analysis through subsequent hydrogel expansion and multiplexed super-resolution imaging. Adapted with permission from 148, copyright 2025 Nature. (D) Schematic of the HNCIB system for concurrent PD-L1 and mRNA profiling in a single EV. Tumor-derived EVs are identified by the fusion of PD-L1-targeted, MB-encoded liposomes, enabling simultaneous detection of surface protein and internal mRNA via TIRFM. Adapted with permission from 149, copyright 2020 American Association for the Advancement of Science (AAAS).

Advances in single-vesicle analysis have yielded engineered platforms for high-throughput, multiparameter profiling. For example, Chen et al. developed a high-throughput, one-pot platform (miR-nSTEV) for in-situ microRNA profiling within single tumor-derived EVs, utilizing orthogonal dual-protein recognition and DNA-barcoded liposome fusion (**Figure 8A**) 146. This approach enabled rapid, multiplexed analysis of six miRNAs via nanoflow cytometry, achieving 100% accuracy in distinguishing prostate cancer patients from healthy donors and demonstrating superior performance over conventional RT-qPCR for non-invasive cancer diagnostics. Complementing this approach for miRNA-focused profiling, other platforms have been engineered to concurrently detect different classes of biomarkers. For instance, employing a microfluidic digital dual CRISPR-Cas (ddSEE) system for concurrent protein and miRNA detection at the single-EV level, this platform enables high-throughput and absolute quantification of EV subpopulations, achieving 92% accuracy in distinguishing breast cancer patients from healthy control (**Figure 8B**) 147. Building upon the concept of high-throughput single-EV analysis, Wu et al. also introduced the RCA-ExM platform, which integrates rolling circle amplification with hydrogel-based expansion microscopy for multiplexed protein and miRNA profiling of individual EVs (**Figure 8C**) 148. This method facilitates high-content analysis by enabling the simultaneous phenotyping of multiple biomarkers on single EVs from numerous clinical samples. Additionally, Zhou et al. introduced a comprehensive platform (HNCIB) that integrates the simultaneous detection of surface proteins and intravesicular nucleic acids on a single, high-throughput chip (**Figure 8D**) 149. This system not only achieves a throughput of up to 384 samples with minimal volume but also successfully applied this multi-analyte approach in a clinical setting, distinguishing lung adenocarcinoma patients from healthy donors by concurrently quantifying the upregulation of PD-L1 protein, PD-L1 mRNA, and miR-21 in individual EVs 149.

### 3.4 Machine Learning Technology

Machine learning (ML) is a subdiscipline of artificial intelligence (AI) that develops models capable of autonomous decision-making through data-driven learning 150, 151. It includes several learning paradigms such as supervised, unsupervised, semi-supervised, and reinforcement learning. Commonly used model architectures encompass neural networks, for example graph neural networks, LSTMs, and transformers, along with classical methods such as random forests, support vector machines, and regression models 152, 153. As a transformative branch of artificial intelligence, ML is increasingly critical for advancing EV clinical translation. By extracting patterns from complex, high-dimensional data, ML provides powerful tools to overcome key challenges in EV research, including deconvoluting heterogeneity, optimizing manufacturing, and deciphering disease-specific signatures for clinical translation.

**Figure 9:**
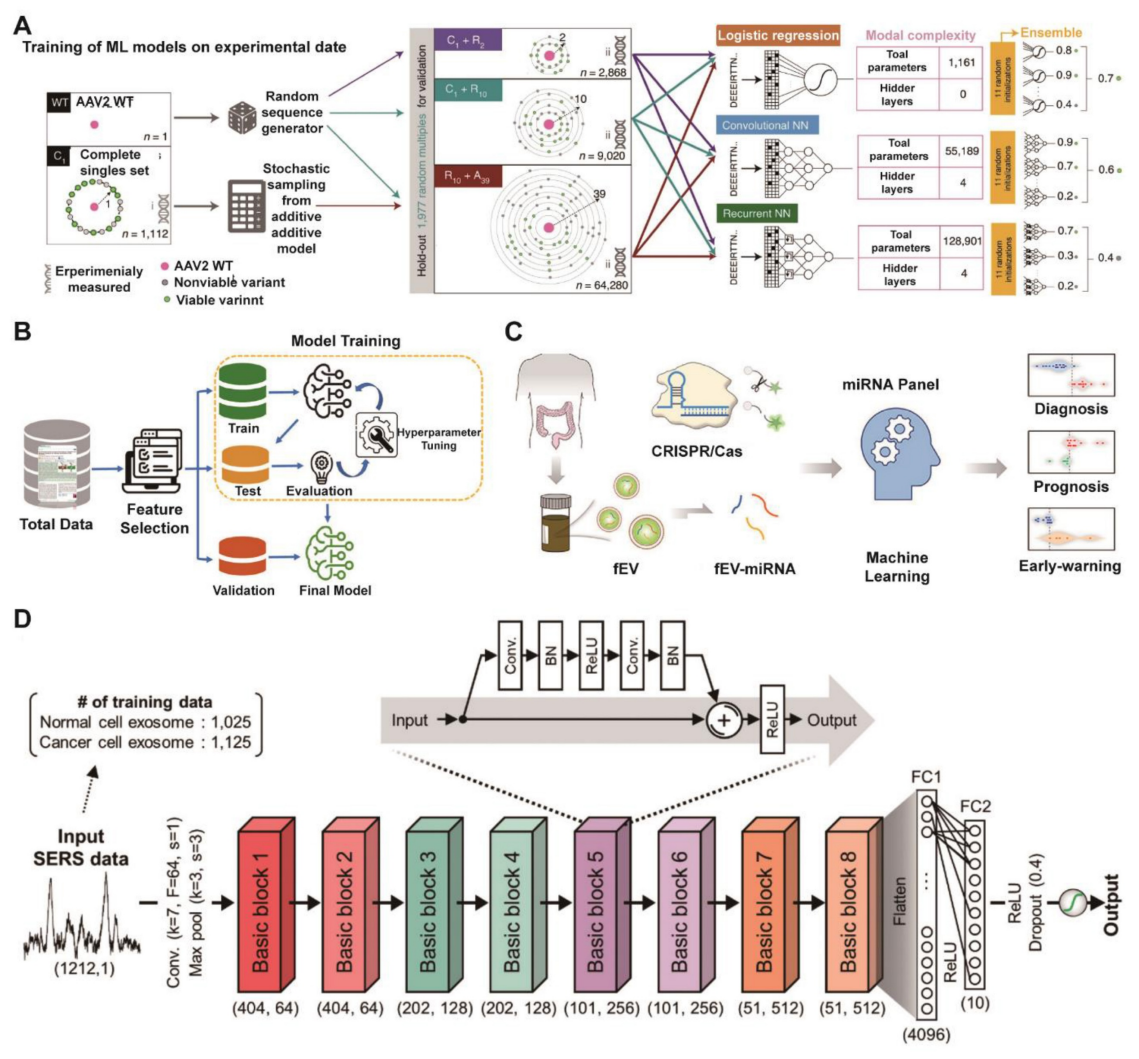
** Machine learning unlocks clinical insights from EV data to accelerate translation.** (A) Workflow for training machine learning models on mutational data, showing integration of complete (C), random (R) and additive (A) sampling strategies into three composite datasets (C1+R2, C1+R10, R10+A39) and performance evaluation of three model architectures (LR, CNN, RNN) with increasing complexity. Adapted with permission from 154, copyright 2025 Nature. (B) Flowchart of the ML-assisted DNA sequencing workflow, predicting nucleotide fingerprint transmission functions to classify unlabeled DNA. Adapted with permission from 155, copyright 2023 American Chemical Society. (C) Schematic diagram for developing a fecal EV miRNA signature as a colorectal cancer biomarker, involving miRNA sequencing, CRISPR/Cas13a detection, and machine learning models. Adapted with permission from 156, copyright 2025 American Chemical Society. (D) Schematic of the ResNet architecture for exosome classification, depicting the complete pipeline from SERS data input through feature extraction blocks to classification output, with key layer specifications and dimensional transformations indicated. Adapted with permission from 160, copyright 2020 American Chemical Society.

The foundational power of ML lies in its ability to rationalize design and analysis processes that are otherwise intractable. This is exemplified in adjacent nanomedicine fields, where ML models have successfully navigated vast biological design spaces. For instance, deep neural networks have been employed to predict the viability of engineered AAV capsids, identifying hundreds of thousands of functional variants and unlocking novel sequence spaces for viral vector design (Figure 9A) 154. Similarly, an ML-aided quantum-tunneling nanopore system has demonstrated high-accuracy classification of DNA nucleotides, establishing a data-driven paradigm for high-precision molecular analysis (Figure 9B) 155. These methodologies provide a transferable computational framework for EV research. Analogous strategies can be adapted to predict the impact of surface engineering on EV targeting, optimize cargo loading protocols, or interpret intricate molecular profiles from single-vesicle analysis technologies. For example, Zhang et al used a linear discriminant analysis (LDA) model to integrate a quadruple-microRNA signature (FEVOR) from fecal EVs. This machine-learning-aided approach achieved a remarkable diagnostic accuracy of 97.4% for colorectal cancer, showcasing the power of computational models to unlock the clinical potential of complex EV-derived biomarkers (Figure 9C) 156.

Additionally, the clinical adoption of circulating EVs for liquid biopsy is hindered by tedious isolation and impurity-prone detection. Addressing this, a study leveraged machine learning (stochastic neighbor embedding) on single-molecule microscopy data to significantly improve analytical accuracy. Addressing this, a study leveraged machine learning (stochastic neighbor embedding) on single-molecule microscopy data to significantly improve analytical accuracy. This approach pinpointed apolipoprotein A1 within EVs as a marker for lipoprotein contamination, demonstrating a powerful strategy for standardizing EV quality control in nanotheranostics 157. Furthermore, ML integrated with thermophoretic aptasensors and surface-enhanced Raman spectroscopy improves the molecular profiling of EVs, enabling early multi-cancer detection and revealing aging-related changes in EV nucleic acid fingerprints 158, 159. Shin et al. also demonstrated a deep learning-based strategy for early-stage lung cancer diagnosis by analyzing the SERS profiles of plasma exosomes (**Figure 9D**) 160. The model, trained exclusively on cell line-derived exosomes, achieved high accuracy (AUC = 0.912) in distinguishing patients from healthy controls, showcasing a promising pathway for non-invasive liquid biopsy without the need for large-scale human clinical data 160. These ML-driven nanoplatforms offer powerful and efficient strategies for advanced disease diagnosis and treatment.

While promising, the application of ML in EV therapeutics faces specific hurdles. Data quality and quantity are paramount; ML models require large, well-annotated, and high-quality datasets, which are still scarce in the EV field due to technological variability and lack of standardization. Model interpretability and generalizability are concerns; complex “black-box” models may perform well on training data but fail on independent datasets from different laboratories or clinical cohorts, limiting clinical utility. There is also a risk of perpetuating biases present in training data. Finally, the integration of ML-derived insights into regulatory submissions requires clear validation frameworks and demonstrable causal links to clinical outcomes, which are still evolving. Therefore, collaborative efforts to build large, shared EV databanks and develop robust, interpretable ML pipelines are essential to fully realize the potential of ML in accelerating EV translation.

## 4. Conclusion

EVs represent a promising class of natural nanoparticles with considerable therapeutic potential, attributed to their innate biocompatibility, efficient biomolecule delivery, and ability to cross biological barriers. However, their translation from bench to bedside remains hindered by substantial challenges in isolation, storage, characterization, biosafety, and quality control. Conventional methods such as ultracentrifugation often suffer from limitations in purity, yield, and scalability, while EV instability under standard storage conditions further complicates clinical deployment. Moreover, the inherent heterogeneity of EVs poses difficulties in precise characterization and dosing, compounded by the absence of universally accepted manufacturing and analytical standards.

To address these bottlenecks, a suite of innovative technologies is emerging to refine EV production and analysis. Advanced bioreactor systems, for instance, enable scalable EV production under tightly controlled conditions, significantly enhancing yield and ensuring critical batch-to-batch consistency. For downstream processing, microfluidic platforms facilitate high-purity isolation with minimal sample loss, overcoming key limitations of traditional methods. Furthermore, single-vesicle analysis techniques are providing unprecedented resolution to deconvolute EV heterogeneity, enabling more precise characterization. Complementing these wet-lab advances, machine learning approaches are being integrated to augment quality control and standardization efforts, offering powerful tools for data integration, pattern recognition, and the acceleration of overall translational workflows.

Ultimately, the clinical realization of EV therapeutics demands focused efforts on standardization, safety assessment, and regulatory alignment. This entails establishing standardized protocols for production and characterization, achieving consensus on isolation methods, quantification metrics, and quality attributes. Furthermore, comprehensive safety and efficacy profiling through rigorous preclinical studies is imperative, covering biodistribution, pharmacokinetics, and immunogenicity. Finally, developing regulatory-ready manufacturing frameworks requires GMP-compliant facilities, scalable processes, and robust potency assays that link EV properties to clinical outcomes. Interdisciplinary collaboration will be key to navigating these final hurdles and translating EV-based therapies from concept to clinic.

## Figures and Tables

**Table 1:** Application of EV-based therapeutics.

Treatment application	Key mechanism	Clinical progress cases	Main advantage
Immunoregulation	EVs carry immune regulatory molecules (e.g., cytokines, miRNAs) Regulate T-cell responses and macrophage polarization Low immunogenicity Modulate inflammatory pathways (e.g., NF-κB, NLRP3) Promote anti-inflammatory macrophage (M2) polarization	Autologous DC-EVs treatment for melanoma (NCT0004586) MSC-EVs for the treatment of COVID-19 (NCT04276987) MSC-EVs for the treatment of GvHD (single case) ExoFlo for ARDS (Phase III, NCT05354141) CD24-EVs for COVID-19 ARDS (Phase IIb, NCT04747574) MSC-EVs for sepsis & acute lung injury (preclinical/clinical)	High biocompatibility Activate innate and adaptive immunity Systemic Immunomodulation with low toxicity Administration via aerosol/nebulization
Drug Delivery	Natural targeting (such as the “immune evasion” effect mediated by CD47) The ability to cross barriers (such as the blood-brain barrier) Engineered targeting (e.g., LAMP2B-RVG, LAMP2B-IL3) Enhanced drug loading via electroporation, incubation, genetic fusion	iExosomes targeting Kras with siRNA (NCT03608631) Paclitaxel EVs inhibit lung metastasis (NCT01854866) Exosomes loaded with STING agonists (exoSTING, NCT04592484) Engineered EVs for chronic myeloid leukemia (CML) targeting IL3 receptor EVs delivering CRISPR-Cas9 for liver cancer	Superior to liposomes (targeting, biological distribution) Deliver various drugs (chemotherapy drugs, nucleic acids, anti-inflammatory agents) Engineering for tissue-specific targeting Protecting cargo from degradation and immune clearance
Regenerative Medicine	EVs transfer functional RNA to promote tissue repair Carry growth factors, anti-apoptotic proteins, pro-angiogenic molecules Modulate oxidative stress and ferroptosis pathways	Umbilical cord-derived MSC-EVs for the treatment of hearing impairment (single case) MSC-EVs for acute liver failure (ALF) & severe acute pancreatitis (SAP) (preclinical/clinical) EV-loaded hydrogels for bone repair (preclinical) AB126 (neural EV) for stroke & neurodegenerative diseases (IND approved 2024)	Avoid the risks of cell therapy (such as carcinogenicity) Promote angiogenesis, reduce fibrosis, enhance cell survival Combining with biomaterials (hydrogels, microneedles) for sustained release

**Table 2:** Critical challenges in EV clinical translation.

Domain	Core Issues	Technical Limitations	Clinical Impact
Isolation	Low purity (co-isolated contaminants) Poor scalability EV heterogeneity	UC/SECmethods compromise integrity/yield Affinity techniques lack scalability No standardized orthogonal workflows	Inconsistent dosing Batch variability Manufacturing bottlenecks
Storage	Instability during long-term storage Structural damage from freeze-thaw cycles	Long-term storage instability at -80°C Lyophilization causes dehydration stress Protectants require optimization	Reduced therapeutic efficacy Limited clinical accessibility
Characterization	Low-resolution bulk analysis Small-EV (<200 nm) detection limits Masked subpopulation heterogeneity	Complex sample prep and prone to artifacts in TEM DLS and NTA with limitations in particle size detection Flow cytometry limited in small-sized EV capture Batch analysis fails to characterize individual vesicles	Molecular mechanism research hindered Functional subgroup identification incomplete
Biosafety	Neurotoxicity risk (BBB crossing) Tumorigenicity controversy Uncertain immunogenicity Lack of long-term toxicity data	Limited toxicokinetic tools Species translation gaps Immune regulation-safety balance difficulty	Regulatory approval complexity increased Risks of long-term use unknown
Quality Control	Absence of EV-specific cGMP Raw material risks (viral/animal-derived) No terminal sterilization	Lack of a dedicated EV quality standard system Microbial control challenges Rigorous process validation	High production costs Regulatory compliance hurdles

## Abbreviations

AEC: Anion exchange chromatography
AFM: Atomic force microscopy
AI: Artificial intelligence
AUC: Area under the curve
BBB: Blood-brain barrier
CTCs: Circulating tumor cells
cGMP: Current Good Manufacturing Practice
ddPCR: Digital droplet PCR
DLD: Deterministic lateral displacement
DLS: Dynamic light scattering
EMA: European Medicines Agency
EOF: Electroosmotic flow
EVs: Extracellular vesicles
FCM: Flow cytometry
FDA: Food and Drug Administration
GLP: Good Laboratory Practice
iSCAT: Interferometric scattering
LDA: Linear discriminant analysis
ML: Machine learning
MSCs: Mesenchymal stem cells
MSC-EVs: Mesenchymal stem cell-derived extracellular vesicles
NTA: Nanoparticle tracking analysis
ODEP: Optically induced dielectrophoresis
PEG: Polyethylene glycol
SAW: Surface acoustic wave
SEC: Size exclusion chromatography
SERS: Surface-enhanced Raman scattering
sEVs: Small extracellular vesicles
SIEVE: Selective isolation of extracellular vesicles
SPARTA: Single-particle automated Raman trapping analysis
TCO: Trans-Cyclooctene
TFF: Tangential flow filtration
TIRF: Total internal reflection fluorescence
TRPS: Tunable resistive pulse sensing
UC: Ultracentrifugation
